# Conservative Management of Negative Pressure Pulmonary Edema: A Case Report

**DOI:** 10.7759/cureus.103213

**Published:** 2026-02-08

**Authors:** Aaditya Jagadish, Jordan M Ross, Brian Gibson

**Affiliations:** 1 General Surgery, Northeast Georgia Medical Center, Gainesville, USA; 2 Research, Northeast Georgia Medical Center, Gainesville, USA; 3 Trauma and Acute Care Surgery, Northeast Georgia Medical Center, Gainesville, USA

**Keywords:** anesthesiology, case report, laryngospasm, negative pressure pulmonary edema, trendelenburg positioning

## Abstract

Negative pressure pulmonary edema (NPPE) represents a less frequently encountered, albeit critical, etiology of non-cardiogenic pulmonary edema, manifesting subsequent to acute upper airway obstruction. It is most often seen in young, healthy adults with strong inspiratory effort, usually triggered by post-extubation laryngospasm. Although the presentation can be dramatic, most patients recover completely when the condition is recognized and managed early. A young adult man underwent a laparoscopic appendectomy under general anesthesia. During emergence, he developed acute laryngospasm while biting down on the endotracheal tube, resulting in strong inspiratory effort against an obstructed upper airway. Shortly after extubation, he produced copious pink, frothy sputum and experienced hypoxemia consistent with NPPE. He was managed conservatively with supplemental oxygen, gentle suctioning, lateral and mild Trendelenburg positioning, and a single dose of intravenous furosemide. His oxygenation improved within hours, and he was discharged home later on, the same day without further complications. Most reports of NPPE describe more severe cases that require reintubation and mechanical ventilation, often with positive end-expiratory pressure or advanced ventilatory modes. In contrast, this case demonstrates that mild NPPE can resolve rapidly with conservative, non-invasive treatment when identified early. The patient's ability to maintain spontaneous ventilation and stable hemodynamics allowed for a measured approach focused on relieving obstruction, improving oxygenation, and promoting alveolar fluid clearance. Early lateral positioning and diuresis likely helped mobilize pulmonary fluid and enhance recovery. This case underscores that NPPE presents on a spectrum, from mild, self-limited episodes to severe respiratory failure, and that treatment should be tailored accordingly. Early recognition and prompt conservative management can lead to rapid recovery in mild NPPE, preventing unnecessary reintubation and the risks associated with mechanical ventilation. Awareness of its variable presentation is essential for safe anesthetic practice.

## Introduction

Negative pressure pulmonary edema (NPPE) is a rare but important cause of non-cardiogenic pulmonary edema that can develop after an episode of upper airway obstruction. It occurs when a patient tries to inhale forcefully against a closed or blocked glottis or an obstructed upper airway, creating high negative intrathoracic pressures. This sudden shift increases venous return and capillary pressure in the lungs, causing fluid to leak into the alveoli and interstitial space [1,2]. Although the overall incidence is quite low, the speed of onset and the potential for rapid deterioration make it a complication that anesthesiologists should always keep in mind [3]. NPPE is most often seen in young, otherwise healthy adults, typically men aged 20-40 who can generate very strong inspiratory efforts [4]. The most common setting is post-extubation laryngospasm, but it can also occur if a patient bites down on the endotracheal tube or develops airway swelling or obstruction during emergence [5]. The presentation exists on a spectrum from mild to severe and can be dramatic, with pink frothy sputum, tachypnea, and hypoxemia, often requiring reintubation or positive pressure ventilation to achieve resolution. More typical, mild presentation can also be mistaken for aspiration or cardiogenic pulmonary edema [6], making quick recognition key to tailoring management strategies and avoiding unnecessary interventions and complications. The presented case underscores the importance of quick recognition and conservative management strategies for the effective management of mild NPPE.

## Case presentation

A young adult man with no prior medical or surgical history presented to the emergency department (ED) with abdominal pain. On arrival, the patient reported that pain started around 7:00 AM in the epigastric and periumbilical regions and eventually migrated to both lower quadrants as the day progressed, prompting him to seek treatment in the ED. The pain was described as sharp and constant, associated with nausea but no vomiting. The patient was afebrile and hemodynamically stable. Abdominal examination revealed generalized tenderness with pain on palpation of McBurney's point. Bowel function was preserved, and he continued to pass flatus. Review of systems was noted only for dysuria. He denied hematuria, abnormal discharge, or sexual activity. Cardiopulmonary, neurological, and musculoskeletal examinations were otherwise unremarkable. Initial laboratory tests were largely normal, except for C-reactive protein (Table 1). 

**Table 1:** Initial laboratory results

Test	Value	Normal range
Neutrophils (absolute)	11.0×10⁹/L	2.0-7.0×10⁹/L
Neutrophils (%)	76%	40-70%
Hemoglobin	14.9 g/dL	13.5-17.5 g/dL (male)
Creatinine	0.73 mg/dL	0.6-1.2 mg/dL
C-reactive protein	2.97 mg/dL (≈29.7 mg/L)	<0.5 mg/dL
Lipase	Normal	0-160 U/L
Urine appearance	Turbid	Clear
Specific gravity	1.033	1.005-1.030
Urine pH	8.0	4.5-8.0
Protein (urine)	Trace	Negative
Blood (urine)	Negative	Negative
Pyuria	None	<5 WBC/HPF

**Figure 1:**
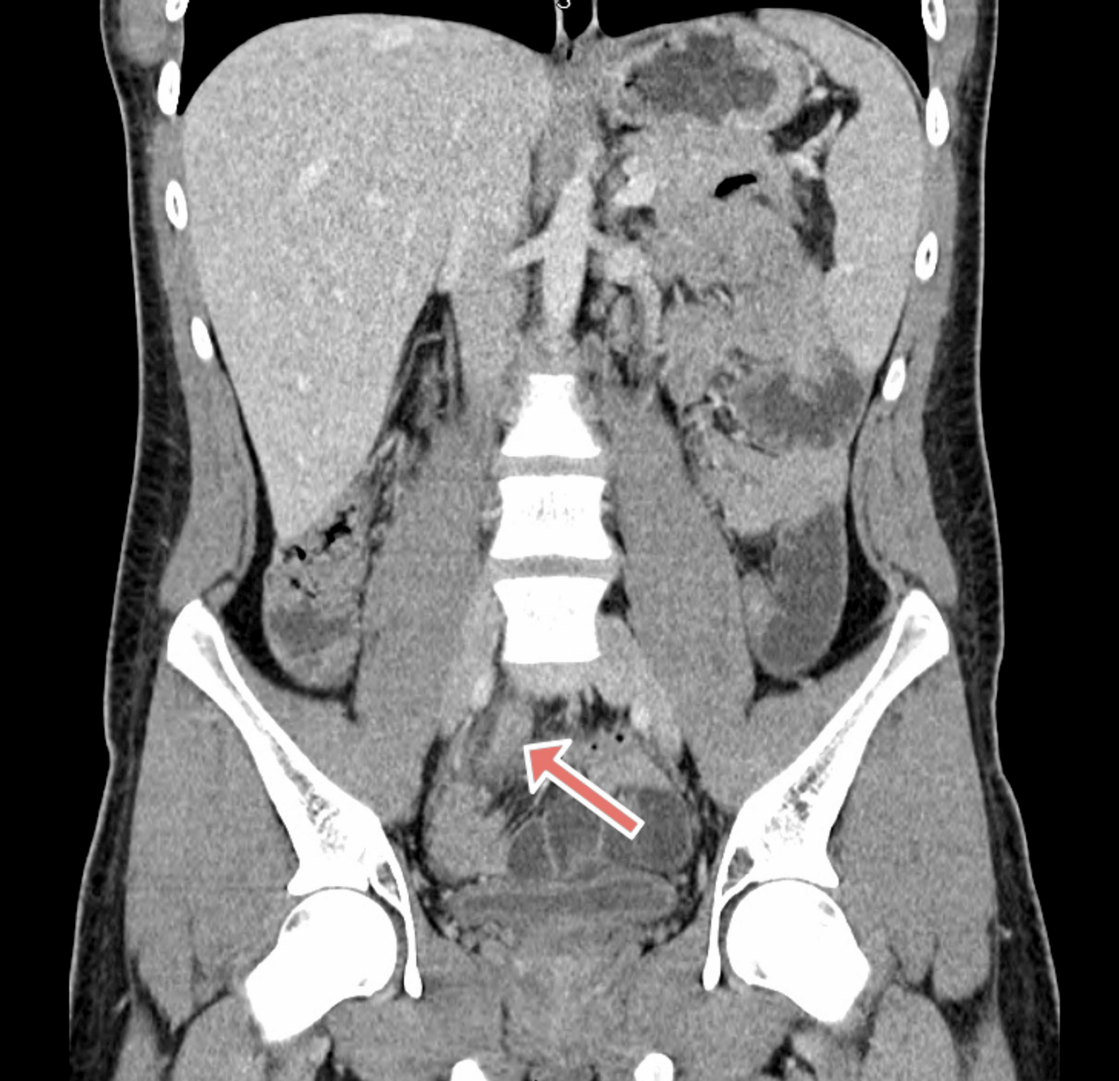
Coronal CT of the abdomen and pelvis with contrast showing appendicitis

CT of the abdomen and pelvis with contrast showed appendiceal wall thickening and peri-appendiceal stranding, consistent with acute appendicitis without perforation or abscess (Figure 1). He was admitted, kept NPO, and started on intravenous piperacillin-tazobactam (4.5 g every six hours) as prophylactic antibiotics. He also received ondansetron 4 mg for nausea and enoxaparin 40 mg subcutaneously every 12 hours for deep vein thrombosis prophylaxis. 

Preoperative assessment 

He was scheduled for a laparoscopic appendectomy under general anesthesia. Preoperative vitals were largely normal: blood pressure of 100/57 mmHg, heart rate of 92 bpm, respiratory rate of 18, and peripheral oxygen saturation (SpO₂) of 99% on room air. Airway exam noted Mallampati class III with adequate thyromental distance, full neck range of motion, and normal mouth opening. Cardiovascular, pulmonary, and neurological exams were normal. He was classified as American Society of Anesthesiologists (ASA) I. 

Intraoperative course 

The patient underwent a laparoscopic appendectomy. General anesthesia was induced with propofol 200 mg, fentanyl 100 mcg, lidocaine 100 mg, rocuronium 50 mg, and esmolol 10 mg. After induction, endotracheal intubation was carried out without any complication. He also received dexamethasone 4 mg and cefazolin 2 g intravenously. Maintenance was with sevoflurane at 1.3 MAC in oxygen (2.2 L/min). Hemodynamics were stable throughout. The case lasted 60 minutes with an estimated blood loss of 10 mL, a urine output of 100 mL, and 1 L lactated Ringer's given. Additional intraoperative medications included fentanyl, phenylephrine, acetaminophen, dexamethasone, dexmedetomidine, and ondansetron. Neuromuscular blockade was reversed with sugammadex 200 mg. 

During extubation, the patient clenched down on the endotracheal tube and developed acute laryngospasm with vigorous inspiratory effort against a closed glottis. The spasm was broken with positive pressure ventilation and jaw thrust, and he was extubated successfully.

Postoperative complication 

Shortly after arrival in the post-anesthesia care unit, the patient required a simple face mask to maintain SpO₂ above 90% and was noted to have a persistent cough with the production of copious pink, frothy, serosanguinous sputum. Though no confirmation imaging was performed, the presentation was consistent with NPPE, and treatment began immediately. He was positioned laterally in Trendelenburg, given supplemental oxygen, suctioned, and treated with intravenous furosemide. Hemodynamics remained stable, and oxygen saturation improved steadily, reaching >95% on room air within hours. This treatment was used given that the presentation of the pathology was not as severe as other cases of NPPE, and the patient began to expectorate the sputum on his own prior to positioning in the Trendelenburg position. 

Postoperative course and outcome 

The remainder of his recovery was uncomplicated. He remained stable with no further hypoxemia, chest discomfort, or respiratory distress. Pain and nausea were well controlled, and his hydration status was adequate. Following a detailed discussion with the patient and his family, he was discharged home later the same day in good condition. Following discharge, informed consent was obtained from the patient for publication. 

## Discussion

In the case described in this report, our patient developed a mild form of NPPE that improved rapidly with simple, conservative management without the need for reintubation or positive pressure ventilation. While no postoperative imaging was performed to directly confirm mild NPPE, all symptoms were consistent with the diagnosis, and the treatment team responded rapidly. Most cases described in the literature involve more severe respiratory compromise that requires airway re-establishment, continuous positive airway pressure, or even invasive mechanical ventilation such as airway pressure release ventilation [7,8]. In this case, the patient was managed with supplemental oxygen, gentle suctioning, lateral and mild Trendelenburg positioning, and a small dose of intravenous furosemide. Within a few hours, his oxygenation normalized, and he recovered completely. This case highlights the range of NPPE severity and management strategies. When the patient is maintaining good respiratory effort and oxygenation, a conservative approach can be both safe and effective. Avoiding unnecessary reintubation for milder cases prevents additional airway trauma, hemodynamic swings, and the risks associated with mechanical ventilation [2,9]. While semi-upright positioning is the established standard to reduce venous return in severe pulmonary edema, Trendelenburg positioning was utilized in this specific instance to facilitate the gravity-assisted clearance of profuse, frothy pink sputum. As the patient remained conscious with a preserved cough reflex, this unconventional positioning prioritized airway patency over preload reduction, allowing the patient to effectively expectorate secretions that otherwise threatened to obstruct the upper airway.

Comparison with previously reported cases 

Most reports of NPPE describe patients who required positive pressure ventilation to achieve recovery. These include cases caused by laryngospasm, upper airway trauma, or obstruction of supraglottic devices [10-12]. In one large review, over 80% of patients required intubation, with recovery taking one to two days on average [1]. In contrast, our patient's rapid improvement and same-day discharge show that NPPE can occasionally resolve much faster when recognized and managed early. The key difference in this case was early identification and intervening immediately at the first signs of hypoxemia and pink secretions, rather than waiting for full-blown respiratory distress. This likely allowed the reversal of NPPE before it became severe. The combination of early positioning, oxygen support, and diuresis may have helped mobilize fluid and improve ventilation-perfusion matching. 

A stepwise approach for mild NPPE 

Based on this experience and existing reports, a stepwise approach may be helpful. Mild cases (SpO₂ >90% on supplemental O₂, stable vitals) may be managed by oxygen therapy, airway clearance, lateral positioning, and cautious diuresis [13]. Moderate cases may be managed by non-invasive ventilation (non-invasive positive pressure ventilation or continuous positive airway pressure) to reduce the work of breathing and promote alveolar recruitment [1]. Severe or worsening cases may be managed by endotracheal intubation with mechanical ventilation, use of positive end-expiratory pressure, or advanced modes such as airway pressure release ventilation [14]. 

The key takeaway is that management should be individualized, not reflexively aggressive [13]. Recognizing that not all NPPE cases require intubation can help tailor treatment to the patient's actual clinical picture. On the contrary, in severe cases, if NPPE is left untreated, it may lead to hypoxemia, heart failure, and even shock [12].

## Conclusions

This case adds to existing literature by showing that mild NPPE can resolve quickly with conservative measures alone, challenging the notion that every case demands invasive support. It also emphasizes the importance of vigilance during emergence and extubation, as early recognition makes a dramatic difference in outcomes. Our approach may be especially relevant in outpatient or resource-limited settings, where avoiding reintubation and intensive care unit admission can significantly improve patient safety and workflow efficiency. While mechanical ventilation remains essential for severe cases, this report shows that timely, thoughtful intervention can lead to excellent outcomes without escalating care unnecessarily. 
